# Epilepsy Detected Using a New Method Based on Volumetric Analysis Results from Brain MR Images

**DOI:** 10.3390/biomimetics11080553

**Published:** 2026-08-04

**Authors:** Orhan Bölükbaş, Harun Uğuz

**Affiliations:** Department of Computer Engineering, Konya Technical University, Konya 42250, Turkey

**Keywords:** epilepsy, anomaly detection, feature selection, negative selection algorithm, brain volume

## Abstract

Epilepsy is a challenging brain disease that requires significant clinical findings. (1) Background: The aim of this study is to improve the success rate of epilepsy detection using a newly developed method by optimizing the high-dimensional dataset obtained from brain MRI images. Standard machine learning models fall short of achieving the desired success in high-dimensional datasets. To achieve this, we aimed to develop an optimized hybrid model by combining the local classification power of the k-Nearest Neighbor classifier and the anomaly detection success of the negative selection algorithm. (2) Methods: Cortical and subcortical brain regions were analyzed to examine volumetric differences. A dataset was created by identifying regions statistically significant for epilepsy. This dataset was then optimized using the Scatter Search Snake Optimization algorithm. The performances of six different machine learning models trained on this optimized dataset were compared. (3) Results: The standard and popular models, SVM (82.70%), kNN (78.70%), RF (69.30%), MLP (73.30%), and NSA (95.89%), demonstrated a detection success rate. In contrast, the proposed hybrid model, kNN-NSA (98.65%), demonstrated a detection success rate. (4) Conclusions: The optimized hybrid kNN-NSA approach, which considers local density in such high-dimensional datasets and tolerates outliers within the self-data, appears to outperform traditional methods. Furthermore, this study has demonstrated that volumetric differences in regions not previously reported in the literature, such as WM-hypointensities, ventral DC, and choroid plexus, may be effective in the decision-making process for diagnosing epilepsy, as they are also found to be significant.

## 1. Introduction

Brain volume and surface area are important in the diagnosis of many diseases in both children and adults. Studies on many diseases, such as autism, ADHD, schizophrenia, multiple sclerosis, epilepsy, and Alzheimer’s disease, highlight changes in brain volume. Volume and surface area are used as important data in the study of brain function [1]. Epilepsy is a disease that affects men and women of all races, in every region of the world, and at a rate of approximately one in 100 people. In approximately half of patients, no specific cause can be found. Epilepsy is a temporary, abnormal electrical impulse in brain cells caused by short-term brain dysfunction [2]. In addition to clinical information, the most important diagnostic tools for diagnosing epilepsy are EEG and neuroimaging. The diagnosis of epilepsy is often difficult to make, and misdiagnoses can occur. EEG is a helpful diagnostic test for diagnosing epilepsy and classifying the underlying epileptic syndrome. However, EEG alone is not sufficient to establish or rule out the diagnosis of epilepsy. Therefore, magnetic resonance imaging is also used [3]. It can be used to identify structural abnormalities that may be the cause of some epilepsy. Magnetic resonance imaging (MRI) is the imaging study of choice for individuals with epilepsy. Structural and functional assessment of the brain through imaging studies is essential for the diagnosis and management of epilepsy. Images should be reviewed by a specialist experienced in the evaluation of patients with epilepsy [4]. In recent years, studies using advanced radiological methods such as magnetic resonance volumetry (MRV) and voxel-based morphometry (VBM) have reported that epilepsy is not limited to the hippocampus; volumetric changes can also be observed in other areas structurally and functionally connected to the hippocampus. Although various studies have examined the factors that may cause changes in brain volume in epilepsy, studies that evaluate brain areas using the MRV technique neuroradiologically and correlate them with volumetric changes are relatively few [5]. Morphometric brain analyses were conducted to examine structural changes in the brains of individuals with epilepsy. These analyses attempted to identify differences by comparing volumetric values of different brain regions with those of healthy individuals. In a literature review in this area, Cablan et al. [6] conducted a study demonstrating decreased volumetric values in frontal and temporal lobe volumetric analyses. Kemmotsu et al. [7] examined the effects of cortical thinning and white matter abnormalities on temporal lobe epilepsy. Rebecca et al. [8] reported decreased hippocampal and cerebellar volumes in their article. William et al. [9] reported decreased cerebral volume. Keller et al. [10] reported decreased thalamic volume. Salmenpera et al. [11] found decreased hippocampus, amygdala, entorhinal cortex, and perirhinal cortex volumes in their study. Kalviainen et al. [12] reported decreased amygdala volume. Bingwei et al. [13] emphasized that hippocampus and amygdala volumes were low in their study. Park et al. [14] reported decreased cerebellar white matter volume in their study. Hutchinson et al. [15] reported a decrease in the anterior corpus callosum, an increase in the posterior corpus callosum, and an increase in the total volume of the corpus callosum. They also emphasized a decrease in gray matter and white matter in the temporal lobe. Cao et al. [16] reported an increase in gray matter in the frontal lobe and a decrease in the thalamus. Andrew and Hal [17] emphasized the importance of the thalamus, brainstem, hypothalamus, and cerebellum regions. Zelco et al. [18] examined a decrease in cortical gray matter, an increase in cortical white matter, and a decrease in subcortical gray matter and the hippocampus. Bernasconi et al. [19] conducted a study indicating a decrease in the amount of gray matter and white matter. Keller et al. [20] conducted a study indicating a decrease in the amount of gray matter. Bonilha et al. [21] reported a decrease in the volume of the thalamus. Kim et al. [22] examined a decrease in the amount of GM. Pulsipher et al. [23] examined the decrease in volume values of the hippocampus, thalamus, caudate, putamen, and corpus callosum. Elts et al. [24] reported a decrease in volumes of the total brain, hippocampus, and amygdala. O’Downer et al. [25] also examined the decrease in corpus callosum volume in their study. Peng et al. [26] reported that the volumes of subcortical gray matter, hippocampus, caudate, putamen, pallidum, thalamus, and amygdala decreased, with only the right pallidum increasing. Keihaninejad et al. [27] reported a decrease in intracranial volume in their study. Briellmann et al. [28] also reported a decrease in intracranial volume.

Anomaly detection is the process of identifying outliers, or rare events, within data that deviate significantly from “normal” patterns. It plays a critical role in cybersecurity (intrusion detection), industry (fault detection), medicine (disease detection), and finance (fraud detection) [29]. One of the most common forms of this problem is conceptualized as the distinction between “self” (normal) and “non-self” (abnormal), inspired by Artificial Immune Systems (AIS) [30]. However, a fundamental challenge that complicates anomaly detection is the phenomenon known as the “curse of dimensionality” [31]. As the number of features in a dataset increases, similarity measures such as the Euclidean distance between data points begin to lose their meaning, and the data space becomes increasingly sparse. This can cause self and non-self-points to become intertwined in space, or the “normal” region to become undefined.

Artificial Immune Systems (AIS) are computational intelligence models inspired by the biological immune system. The Negative Selection Algorithm (NSA) is a central component of AIS. NSA is based on the principle of generating a set of detectors that first define the “Self” and detect anything that does not match this defined set as a “non-self” or anomaly. This adaptive learning mechanism is quite powerful for anomaly detection, especially when the self-set can be clearly defined [32].

Improving the detection success of a Negative Selection Algorithm (NSA) is a multifaceted process that requires addressing the algorithm’s inherent weaknesses and optimizing the quality of the detector set. Research indicates that hybrid architecture and meta-heuristic optimization techniques offer the most effective solutions for this purpose. Especially on high-dimensional datasets, NSA, a distance-based algorithm, suffers from the “Curse of Dimensionality”, a condition in which distances between data points become meaningless and performance suffers. One of the biggest constraints on NSA performance is the “holes” that arise when the detector set does not perfectly cover the core space. These holes cause non-self (anomaly) samples to be incorrectly classified as self, decreasing the detection rate (DR). Feature Selection: A critical preprocessing step to improve NSA detection success is selecting the optimal feature subset. This reduces the influence of irrelevant or noisy features.

On the other hand, the k-Nearest Neighbor (kNN) algorithm functions as a local classification and anomaly scoring method based on data density [33]. The anomaly score of a test sample is related to its distance to its nearest neighbors or the density of these neighbors.

Building a hybrid kNN-NSA model aims to combine the strengths of the two models. NSA creates a global exclusion region around the Self cluster, while kNN is sensitive to local data distribution. This hybrid approach can improve classification accuracy by combining the overall coverage efficiency of NSA with the ability of kNN to detect local anomaly deviations. This integration is important because ensemble models provide consistent performance improvement over traditional models such as Random Forest or Support Vector Machines.

In this study, a dataset was created by volumetrically analyzing brain MRI images of healthy individuals and those diagnosed with epilepsy, selecting statistically significant regions. Using this dataset, we measured the success of the optimized hybrid kNN-NSA algorithm in detecting epilepsy. With the dataset on which quality optimization was performed with the SSSO algorithm, the model achieved 98.67% detection success and 98.45% accuracy success with the hybrid kNN-NSA algorithm.

In summary, to increase the detection success of NSA, the most suitable model for epilepsy detection was determined by applying the most effective strategy that eliminates the noise and dimensionality problem by selecting features with SSSO [34], which is a meta-heuristic optimization, and determines the detector formation at the most appropriate level by creating a hybrid structure with kNN.

### 1.1. Related Work

This study examines the diagnostic performance of machine learning algorithms by reviewing studies in epilepsy. A brief overview of commonly used machine learning approaches and the need for further application of machine learning techniques in epilepsy are presented. With increasing computational capabilities, the availability of effective machine learning algorithms, and the accumulation of larger datasets, clinicians and researchers will increasingly benefit from familiarity with these techniques and the significant progress already being made in their application in epilepsy.

Machine learning utilizes statistical and computer science principles to develop algorithms that can improve performance by interpreting data rather than explicit instructions. In addition to widespread use in image recognition, language processing, and data mining, machine learning techniques have gained increasing interest in medical applications, from automated imaging analysis to disease prediction. Classification studies have also been conducted to identify patients with epilepsy using values obtained from morphometric brain analyses. A literature search revealed that very few studies have been conducted in this area. Cantor-Rivera et al. [35] achieved an 88.9% success rate in identifying epilepsy patients using SVM. Rudie et al. [36] achieved an 81% success rate using the SVM classification tool in their study. Kodipaka et al. [37] found 80.65% success with SVM in their study based on volume values. Keihaninejad et al. [27] achieved 89% classification success with SVM in their study. Torres-Velázquez et al. [38] classified epilepsy disease with 71.43% success with a multi-channel deep neural network method in their study. Chen et al. [39] achieved 93.3% classification success in mesial temporal lobe epilepsy with the SVM algorithm. John Del Gaizo et al. [40] achieved 82.0% success using the SVM algorithm on diffusion MRI data. Svyatoslav Vergun et al. [41] achieved 90% success in their classification study using the naive Bayes algorithm. Zhou et al. [42] achieved 84% success with the SVM algorithm in their study titled “Machine learning for detecting mesial temporal lobe epilepsy by structural and functional neuroimaging.” Ghazal Sahebzamani et al. [43] achieved 94% success with the SVM algorithm in their study titled Machine Learning Based Analysis of Structural MRI for Epilepsy Diagnosis.

Most studies on epilepsy detection use EEG signal data. However, studies using MRI image data are quite limited. We aim to contribute to the literature with this study.

### 1.2. Contribution of the Study to the Literature

The main contribution of this work is to combine the powerful local classification capability of kNN with the robust anomaly detection mechanism of NSA in an optimized framework. The success of the model, especially in challenging domains such as the analysis of high-dimensional biomedical data, depends on the integration of its three underlying layers. The proposed hybrid architecture for this purpose is as follows:

1. Meta-Heuristic Feature Selection: To address the high-dimensionality problem, Scatter Search Snake Optimization (SSSO) is used to identify the most discriminative feature subset.

2. NSA-Based kNN Optimization: Using the k Nearest Neighbor Algorithm (kNN), the NSA optimizes the radius of the detectors, maximizing specificity and reducing FPR/time to clinically acceptable levels.

3. Patient-Normal Discriminatory Power: This provides a system capable of performing the challenging patient-normal discrimination of a dataset obtained from MR images with high reliability.

## 2. Materials and Methods

**Figure 1:**
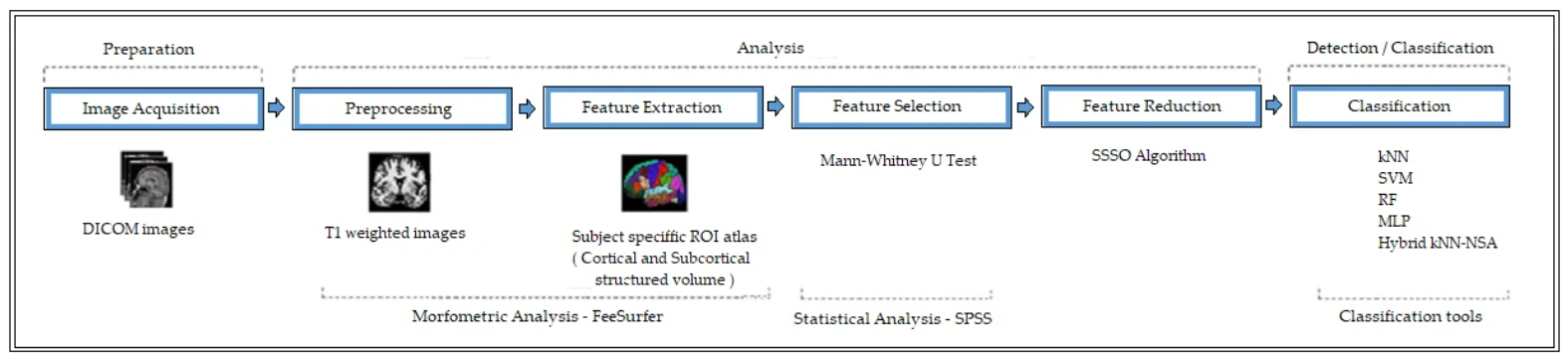
Process steps.

The process steps of the model developed in this section are discussed as shown in Figure 1.

### 2.1. Data Acquisition

The data used in this study were obtained from a publicly available database distributed for research purposes within the scope of the UCI Machine Learning Repository. Additionally, the uniquely created EpilepsyMRI dataset was used. The EpilepsyMRI dataset consists of information related to temporal lobe epilepsy, a subtype of epilepsy.

In this study, MRI images of 121 healthy individuals and 75 individuals diagnosed with epilepsy were obtained from the MRI Service of Aksaray University Aksaray Training and Research Hospital, in accordance with the Ethics Committee Report. The images are in DICOM format and were obtained retrospectively from the neurology clinic archives. In the preprocessing phase, T1-weighted images were used by examining DICOM images taken in sagittal, coronal, and axial positions in T1, T2, and Flair sequences. MRI protocol: MRI morphometric examination was performed using a 1.5T Magnet (Philips Intera, Amsterdam, the Netherlands) device and a standard head coil. A 3D FFE (fast field echo) sequence was obtained using the interhemispheric line for area measurement in the T1-weighted coronal plane, and the following technical factors were applied: TR (repetition time) = 25 msec, TE (echo delay time) = 5 msec, FOV (field of view) = 130 × 160, matrix = 224 × 224, flip angle = 30 degrees, and slice thickness = 0.7 mm for volume, with a gap = 0.

### 2.2. Preprocess

The preprocessing phase involved reviewing CDs of MRI images from individuals obtained from the Neurology archives and preparing them for the FreeSurfer program. For this purpose, DICOM images taken in sagittal, coronal, and axial positions using T1, T2, and Flair sequences were analyzed in the viewer program. Appropriate and clean T1-weighted images were selected.

### 2.3. Feature Extraction

**Figure 2:**
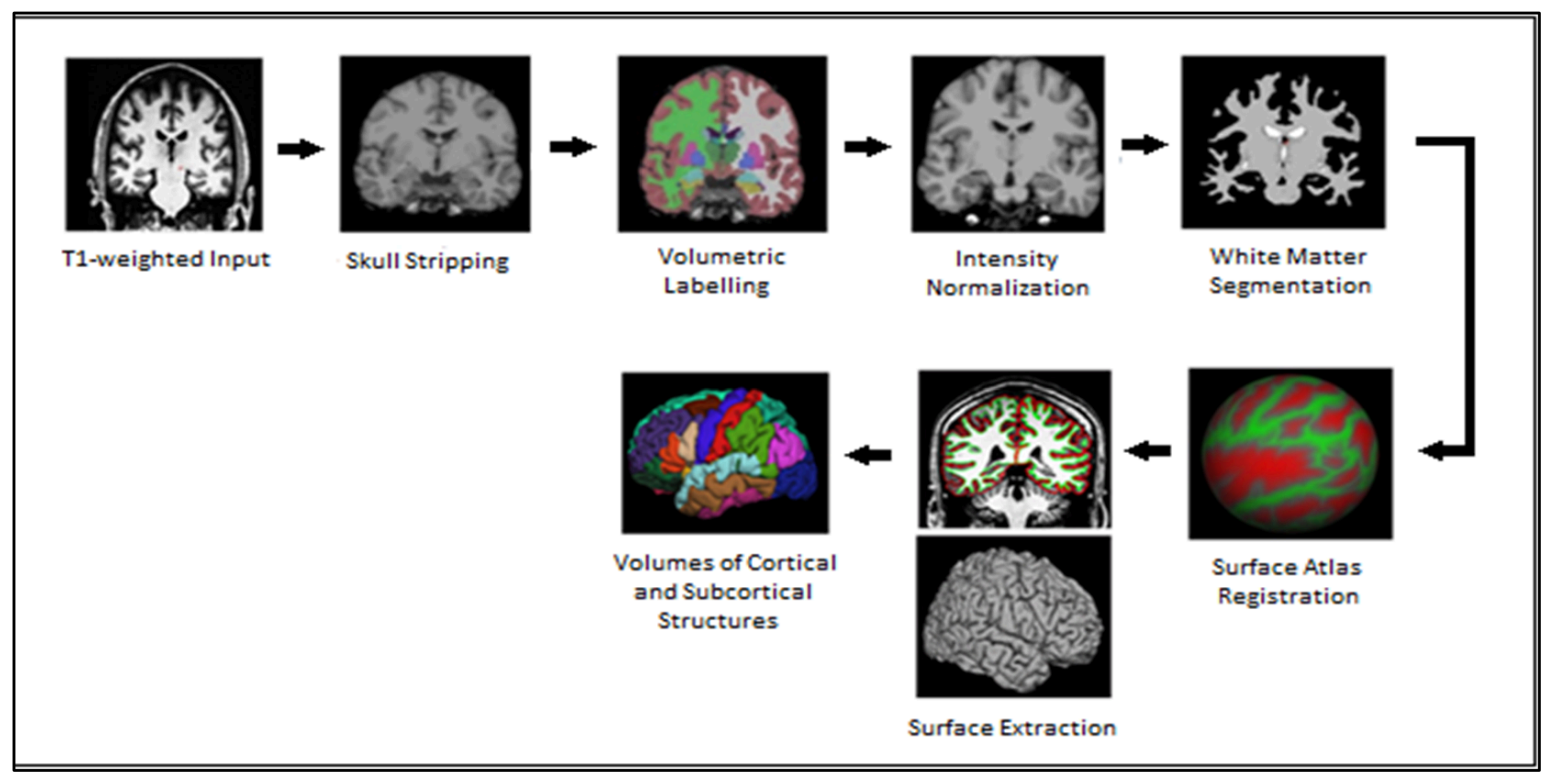
Feature extraction process steps with FreeSurfer Program [44].

The T1-weighted images selected after preprocessing were analyzed using the FreeSurfer [44] program, which is installed on the Linux-based (Ubuntu) operating system. The tissue and brain regions were separated from the selected MR images according to the procedures described by Fischl et al. [45], and the tissue portion was extracted. Cortical and subcortical regions were extracted along with their volumetric values as a result of the analysis performed with the FreeSurfer program. This process automatically segments gray matter, white matter, and subcortical thicknesses, providing reliable volumetric values. The feature extraction process is performed by following the steps shown in Figure 2.

As a result of the analysis conducted with FreeSurfer, regions with volume values were identified as attributes of the dataset prepared for this study. These regions are listed in Table 1.

### 2.4. Feature Selection

When performing classification studies on large datasets, one of the most crucial steps is feature selection. The presence of many features in a dataset increases the training and testing time of machine learning algorithms. Furthermore, some machine learning algorithms are sensitive to redundant features, which can lead to performance degradation. Therefore, feature selection is a frequently used operation in classifier systems. Feature selection is generally performed in two ways: filtering and wrapper methods [46]. In filtering methods, features are ranked according to a selected criterion, and those with the highest ranking are selected. In wrapper methods, the classifier is used as a black box. The classifier performance values of the selected feature sets are checked, and feature sets with high success rates are selected [47]. In this article, statistical tests are used as the filter feature selection method. The relevant tests were performed in SPSS 22.

The method and tests used to analyze data statistically are important because it creates assumptions and hypotheses that will help examine the effects of independent variables on dependent variables. To do this, the data are checked for parametricity before being analyzed. If the group variances are equal and the data have a normal distribution, it is parametric. Otherwise, it is nonparametric. The *t*-test is used in the parametric method, and the Mann–Whitney U test is used in the nonparametric method. The Mann–Whitney U, a nonparametric hypothesis test, is used to investigate whether there is a significant difference in the median between two sample groups [48].

In this study, nonparametric hypothesis testing was used because the data were not normally distributed, with kurtosis and skewness values between −1 and +1, and group variances were not equal. The Mann–Whitney U test, a nonparametric test, was used. The Mann–Whitney U test is a nonparametric test that is an alternative to the independent samples *t*-test. This test is used to examine the mean difference between two independent groups from similar populations and to determine whether there is a difference or equality between the groups. The classification or detection performance of the attributes (regions) was found to be significant because of this test. Using this test, the significance of the regions between the patient and control groups was examined (at a 95% confidence level).

### 2.5. Feature Subset Selection (Feature Reduction)

Feature subset selection is the selection of a smaller subset of features from a larger set of features. This avoids complex computations and makes classifiers work much faster [49]. Sometimes, we try to reduce the size of the feature subset, while other times we try to increase the prediction accuracy. Essentially, feature subset selection aims to create a subset that most accurately represents the dataset by eliminating unnecessary features. In other words, it aims to obtain the minimum number of features while increasing classification accuracy [50].

**Figure 3:**
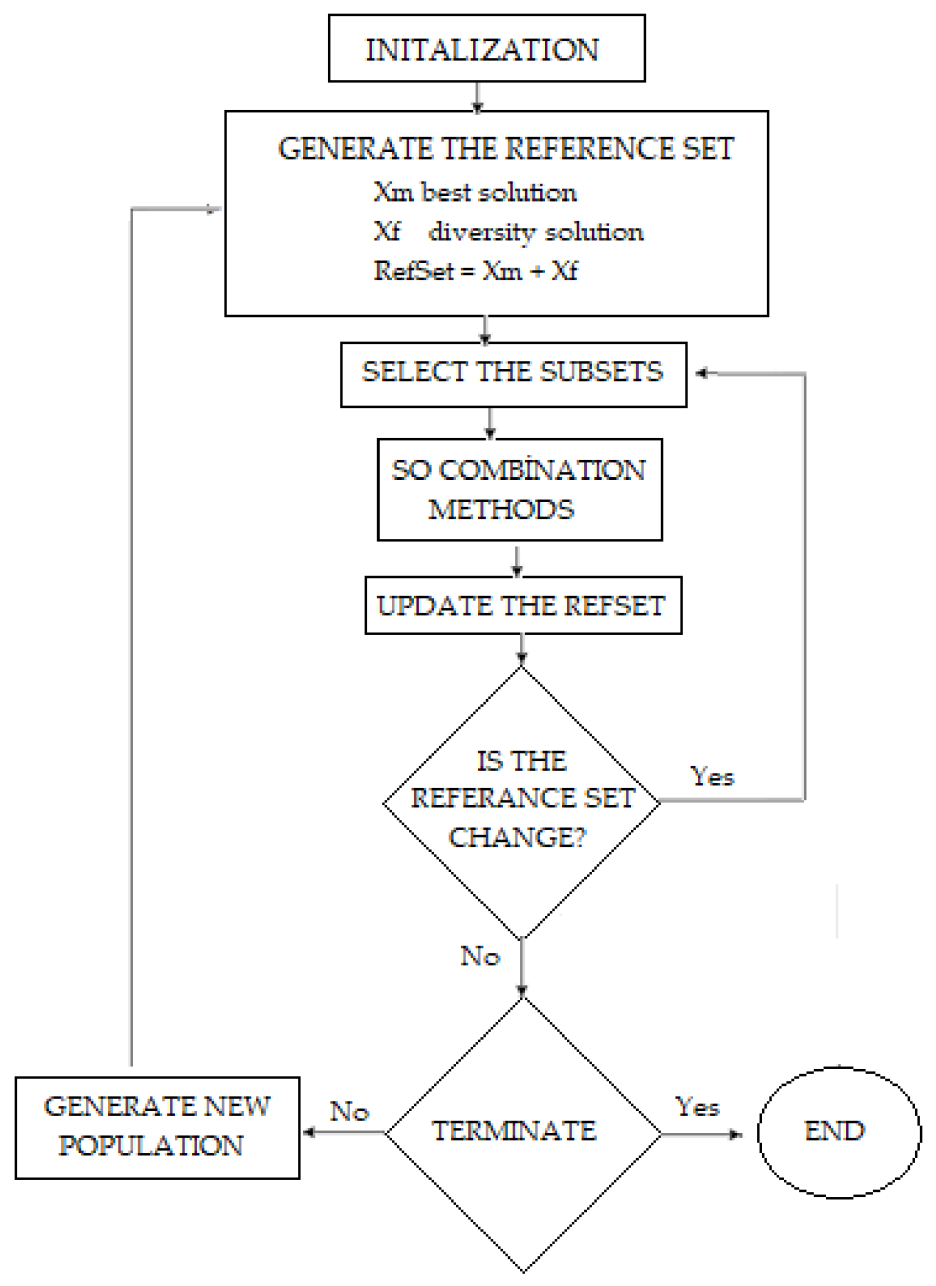
SSSO flowchart [34].

In this study, to improve the performance of the model, the subset selection of the attributes obtained from the MWU statistical test was performed using the SSSO algorithm developed by Bölükbaş et al. [34]. The flowchart of the SSSO algorithm is given in Figure 3.

### 2.6. Machine Learning Algorithms

To test the success of epilepsy detection, machine learning algorithms such as Support Vector Machines (SVMs), k-Nearest Neighbor (kNN), Random Forest (RF), Multilayer Perceptron (MLP), Negative Selection Algorithm (NSA) and Hybrid kNN-NSA algorithm were used.

#### 2.6.1. k-Nearest Neighbor (kNN) Algorithm

The k-Nearest Neighbor method is one of the supervised learning methods used in classification problems. In this method, the similarity of the data to be classified to the normal behavior data in the learning set is calculated, and then the data are assigned to classes based on the average of the k-closest data points and a specified threshold value. The key is that the properties of each class are clearly defined in advance. The method’s performance is influenced by the number of k-nearest neighbors, the threshold value, the similarity measure, and the sufficient number of normal behaviors in the training set. For more information on k-NN, see Zhao et al. [33].

#### 2.6.2. Support Vector Machine (SVM) Algorithm

Support Vector Machines have been successfully applied to numerous classification problems and have earned their place in the literature as one of the most effective machine learning algorithms with high generalization performance. The most significant advantage of Support Vector Machines is that they transform the classification problem into a quadratic optimization problem and solve it. This reduces the number of operations involved in the learning phase and enables faster solutions compared to other techniques/algorithms. Kernel function selection and parameter optimization play a crucial role in the application of Support Vector Machines to solving classification problems for various datasets. The performance of kernel functions can vary depending on the dataset and the problem. For more information on SVM, see Gargia et al. [51].

#### 2.6.3. Multilayer Perceptron (MLP) Algorithm

Multilayer Perceptron neural networks consist of an input layer containing input neurons, an output layer containing output neurons, and one or more hidden layers. Because additional layers are added between the input layer and the output layer to address nonlinear problems, the network architecture is multilayered. Determining the number of hidden layers in neural network architecture is crucial. Unlike creating input and output layers, network architecture begins without prior knowledge of the number of hidden layers. Typically, when creating network architectures, the appropriate number of hidden layers is determined through trial and error. For more information on MLP, see Güler et al. [52].

#### 2.6.4. Random Forest (FR) Algorithm

It is an algorithm that increases the classification rate by generating multiple decision trees during the classification process. The Random Forest method consists of a collection of Classification Trees or Regression Trees, depending on the desired number of trees. Randomly selected decision trees are combined to form a decision forest. It performs well on datasets containing categorical variables with many variables and class labels, missing data, or unbalanced distributions. Therefore, Random Forest is one of the most widely used ensemble methods. The basic idea behind the method is to generate ensembles using a randomly selected subset of predictor trees from many trees. For more information, see the work of Breiman [53].

### 2.7. Developed Machine Learning Model

#### 2.7.1. Negative Selection Algorithm (NSA)

Foreign cells in the innate immune system are recognized by two types of lymphocytes, B and T cells, produced in the bone marrow. Both cells are produced in the bone marrow. The T cells then undergo a process called negative selection in the thymus. During this stage, T cells that do not match the body’s own cells are released from the bone marrow.

**Figure 4:**
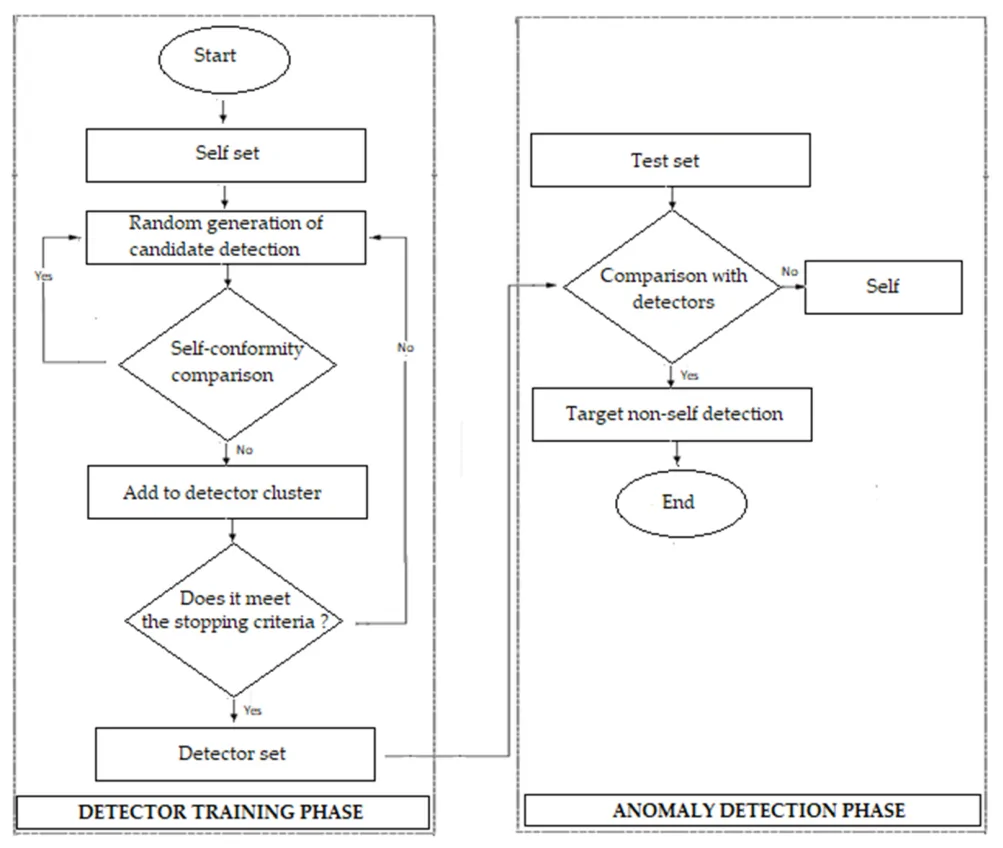
Flowchart of training and testing stages of standard negative selection algorithm.

The remaining cells are destroyed here. This way, all surviving T cells will recognize non-self-cells. This elimination of T cells protects the immune system from attacks by the body’s own proteins. By exploiting this property of the innate immune system, an anomaly detection algorithm based on negative selection of T cells has been developed. This algorithm, called negative selection, has been used for computer security. The most important feature of this algorithm is that it stores information about the unknown pattern set for the system using the available eigen sets and can be used for pattern recognition. The detector generation and error detection flowchart of the negative selection algorithm is shown in Figure 4.

As shown in Figure 4, the set of patterns to be preserved is first determined and called the ‘self-set.’ Based on the negative selection algorithm, a set of detectors responsible for identifying elements that do not belong to the ‘self-set’ is created. The negative selection algorithm eliminates detectors by comparing them with the training set of randomly generated detectors. This comparison is performed using similarity functions known as affinity. The affinity function, based on the Euclidean distance measure, is shown in (1) and (2). Equations (1) and (2) show the comparison calculation of the i. element for the n-length training dataset (core dataset) and the randomly generated detector set.

(1) $$Dis\left(D,E\right)=\sqrt{\sum_{i=1}^{n}{\left(D_{i}-E_{i}\right)}^{2}}$$

(2) $$A=Dis\left(D_{i},E_{n}\right)-\epsilon$$

Here, the affinity value A is determined by the values of ε (threshold value) and dis (distance). If the value of A is less than zero, the randomly generated detector is eliminated. Otherwise, the detector is added to the real detector set.
**Require:** Generate pre-defined number of constant-sized detectors *S*       : set of self-samples *m*      : number of detectors *rs*      : self-radius 1: *D* ← *∅* 2: **repeat**              {repeat until re-defined number is reached} 3:         *x* ← random sample from [0, 1]*n* 4:       **for all** every *si* in *S* = {*si*, *i* = 1, 2, ⋯ } **do** 5:                  *d* ← Euclidean distance between *si* and *x* 6:                    **if**
*d* ≤ *rs*
**then** 7:                            go to 2                  {When recognized as self, try a different point} 8:                  **end if** 9:       **end for** 10: *D* ← *D* ∪ {*x*}        {If not matched by any self-samples, add to the detector set} 11: **until** |*D*| = *m*

The comparison between the anomalous and normal cases is similar. After creating the detector set, the next step of the algorithm consists of monitoring the system for the presence of non-self patterns. In this case, a test set is created. This set consists of the self-set and the non-self set, or an entirely new non-self set. For all elements of the sensor set corresponding to non-self models, a match is checked to see if it recognizes an element of the test set. If there is a match, then a non-self model has been recognized, and the reaction should begin. In non-self detection, the result action evaluation varies depending on the problem and is outside the pattern recognition scope of the negative selection algorithm. To construct the NSA’s detector set, the algorithm steps developed by Forrest and Perelson [30] were followed. Below are the process steps showing the detector training phase of the negative selection algorithm [32].

#### 2.7.2. Developed Hybrid kNN-NSA Model for Epilepsy Detection (Hybrid kNN-NSA)

The model referred to as hybrid kNN-NSA is an anomaly detection approach that combines the Negative Selection Algorithm (NSA) from the field of Artificial Immune Systems with the optimized k-Nearest Neighbor (kNN) algorithm.

Anomaly detection is the process of identifying outliers or rare events within data that deviate significantly from “normal” patterns. It plays a critical role in cybersecurity (intrusion detection), industry (fault detection), and finance (fraud detection) [29]. One of the most common forms of this problem has been conceptualized as the distinction between “self” (normal) and “non-self” (abnormal), inspired by Artificial Immune Systems (AIS) [31].

However, the size of the problem or the number of features in the dataset makes anomaly detection difficult [32]. As the number of features (dimensions) in the dataset increases, similarity measures such as the Euclidean distance between data points begin to lose their meaning, and the data space becomes increasingly sparse. This causes self- and non-self-points to become intertwined in space, or a clear boundary of the normal region cannot be drawn.

In this study, disease detection was performed by reducing a high-dimensional dataset to 13 dimensions (features) using the SSSO algorithm. It is known that standard machine learning models often fail on such high-dimensional datasets. The aim of this hybrid model is to improve the success of epilepsy detection by combining the logic of the Negative Selection Algorithm (NSA) with the Positive Selection philosophy and the selection approach of the k-Nearest Neighbor (kNN) algorithm. It also proposes a hybrid approach that models the inside of the “normal” region (Positive Selection), as opposed to the Negative Selection (NSA) algorithm’s philosophy of modeling the outside of the “normal” region, but utilizes the local density estimation capabilities of kNN.

In high-dimensional datasets, it can be said that the performance of the model depends on optimizing the algorithm with the correct hyperparameters according to the structure of the data rather than the selected algorithm.

This model is used to detect “Epilepsy” (Non-Self) data that is not like the dataset defined as “Normal” (Self). The logical flow and process steps of this hybrid model are as follows:
**Stage 1: Training (Detector Generation and Optimization)** At this stage, the model learns what “normal” data are and prepares “detectors” to catch anomalies.
**Getting Started (Data Collection):**○Collect a training set () containing only normal (Self) data.**Detector Production (NSA Phase):**○Generate random candidate detectors () in the feature space. (A detector can be thought of as a center and radius to cover the “anomalous” area.)**Maturation/Selection (NSA Stage):**○Retrieve each generated candidate detector ().○Check if this detector matches any data point in the “Self” dataset () (i.e., measure if it is closer than a specified distance).○**IF** detector “Self” matches (overlaps) with a data point -> discard this detector (because it should not label normal data as abnormal).○**IF** detector “Self” does not match the data -> Add this detector to **the mature detector set ().****Cycle:**○Repeat steps 2 and 3 until a sufficient number of mature detectors are obtained (or enough to provide adequate coverage).○Problem: This method may not be able to generate detectors in the small holes between the “Self” data, leading to false negatives (missed anomalies). This is where kNN comes in.**Detector Improvement (kNN Hybrid Stage):**○This step can vary across models. A common approach is to use kNN to refine data that is labeled “Self” by the NSA but lies far from the dense regions of the “Self” set (i.e., likely to be in “holes”).○kNN is applied for suspicious points classified as “Self” by NSA (i.e., not matching any detector).○If the majority of the k-Nearest Neighbors of this point are “Self”, the point is confirmed as “Self”.○If its neighbors are not “Self” (or are very sparse), this region can be marked as a “hole” and special detectors can be added there or kNN classification can be used directly in the testing phase.
**Stage 2: Testing (Epilepsy–Anomaly Detection)** Once the model is trained, it classifies whether the newly incoming data are normal or indicative of epilepsy.
**Get New Data:**○Get new test data () to be classified.**Primary Classification (NSA):**○Does the test data () match any detector in the “Mature Detector Set” ()?○**IF** matches -> Label the data as **EPILEPSY (Non-Self)** and finish the process.○**IF** not match -> Data may be “Self” or in a “hole”. Go to step 3.**Secondary Classification (Validation with kNN):**○For test data () found by NSA as “Normal” (no match):○Find the k nearest neighbors to point () in the original “Self” training set.○Calculate the average distance to these k neighbors.**Final Decision:**○Is the calculated average distance greater than a predetermined **threshold**?○**IF YES (Distance > Threshold):** The data point is far from the normal data density region (located in a “hole”). Label the data as **EPILEPSY**.○**IF NO (Distance <= Threshold):** The data point is close to the region where normal data are concentrated. Label the data as **NORMAL (Self)**.


Pseudo code example for a Negative Selection Algorithm (NSA) + kNN hybrid approach:
text
Input:
   SelfSet = normal instances
   DataSet = new instances to classify
   k = number of neighbors
Step 1: NSA Detector Generation
   Detectors = ∅
   while Number of Detectors < N:
      candidate = generate random sample
      if candidate does not match SelfSet:
         Detectors ← Detectors ∪ {candidate}
Step 2: Pre-Filtering with NSA
   For each sample in DataSet:
      if sample matches any Detectors:
         sample.label = “non-self”
      else:
         sample.label = “self”
Step 3: Detailed Classification with kNN
   For each sample in DataSet where sample.label == “non-self”:
            neighbors = find the k nearest neighbors (in SelfSet ∪ NonSelfSet)
            class = majority_vote(neighbors)
            sample.final_label = class
Step 4: Output
   For each sample:
      If “self” → normal
      If “non-self” → anomaly type with kNN result

This flow represents a hybrid architecture that aims to solve the “hole” problem of NSA by combining the fast anomaly detection capability of NSA (detector matching) with the density-based boundary detection capability of kNN (distance measurement).

## 3. Results

This chapter includes an introduction to the experimental setup, problem examples, and a report on the experimental results.

### 3.1. Experimental Setup

All operations were performed on a machine with a 2.8 GHz CPU, 20 GB RAM, and Windows 11 and Ubuntu operating systems. Feature extraction was performed with FreeSurfer, and statistical analyses were performed with SPSS 22 [54]. Machine learning algorithms were implemented using Matlab 2024 and Python 3.0 programming languages.

### 3.2. Performance Measures

Since our dataset includes epilepsy patients and healthy controls, we apply binary classification. The metrics listed in Table 1 are Precision (Prec) to measure the accuracy of the detections, Recall (Recall) to detect anomalies, F1 score to avoid incorrect model selection in unevenly distributed datasets, and Accuracy (Acc) to measure the model’s success. They are used to evaluate the performance of classifiers.

### 3.3. Results of the Feature Extraction Method

In this study, we used the EpilepsyMRI dataset, which we created to identify patients with epilepsy. The MRI images used in this dataset were obtained in retrospective DICOM format from the Aksaray University Aksaray Training and Research Hospital MRI Neurology Archive. These images, belonging to 75 epilepsy disease and 121 healthy individuals, were subjected to structural morphological brain analysis using the FreeSurfer [44] program. Demographic information obtained from the healthy and patient groups is shown in Table 2. This analysis yielded the volume values for the 50 brain regions shown in Table 3. In this study, these extracted regions were used as the raw features of the dataset. These numerical values were used to create a raw dataset. This dataset was used in the training and testing phases to determine the model’s performance.

### 3.4. Results of the Feature Selection Method

The 50 regions shown in Table 4 were subjected to the independent samples Mann–Whitney U test, a statistical test for significance in terms of epilepsy. Because the test revealed a significant volumetric shift in the cortical and subcortical brain regions of epilepsy patients, the volumetric differences in these regions are important for the diagnosis of epilepsy. The Mann–Whitney U test findings are shown in Table 4. As a result of the analysis, 35 regions were found to be significant at the *p* < 0.05 level for identifying epilepsy. These regions are marked in red in Table 4. These 35 regions selected by the Mann–Whitney U test will be considered features of the dataset to be used in disease detection. These regions are the ones that will distinguish between people with epilepsy and those without. In our dataset, these regions will represent the features. This created dataset was named MWU35.

### 3.5. Results of the Feature Subset Selection (Feature Reduction) Method

The feature subset selection performance of the SSSO algorithm developed by Bölükbaş et al. [34] is given in Table 5. As can be seen in this table, the performance of SSSO is better compared to other optimization algorithms.

The highest accuracy value was obtained using the PRO method (87.31%), while the proposed SSSO method achieved a performance quite close to that of PRO (87.12%). In terms of error rate, the PRO method achieved the lowest error rate (12.69%), while the SSSO method produced a competitive result with an error rate of 12.88%. In terms of precision, the PFA method achieved the highest value at 95.67%, while the SSSO method achieved a precision value of 95.51%, which is quite close to the performance of PFA. In terms of accuracy metrics, the MRFO method yielded the best result (86.01%), while the SSSO method produced a successful accuracy value of 85.73%. Considering the F1 score as a balanced combination of sensitivity and specificity, the PRO method provided the highest performance (90.41%), while the SSSO method achieved a result (90.36%) quite close to that of PRO. However, in terms of specificity, the SSSO method showed a relatively weak performance compared to other methods, with a value of 73.75%. This may indicate that the method has some limitations in distinguishing negative examples. Overall, the SSSO method demonstrates strong performance, particularly in terms of accuracy, precision, and F1 score, proving to be an effective solution to feature selection problems [34].

Regions identified as significant for epilepsy using the Mann–Whitney U statistical method were selected as features of the dataset. Feature subset selection was performed on this dataset using the SSSO algorithm to improve model performance. The results are shown in Table 6.

By optimizing feature selection using the SSSO algorithm in the dataset, 35 features were reduced to 13. The dataset created with the SSSO algorithm is named SSSO-13. The model’s performance because of this feature reduction is given in Table 6.

### 3.6. Parameters Used in Machine Learning Algorithms

After preprocessing techniques, our data was trained using 10-fold cross-validation. Parameters in Support Vector Machines (SVMs), k-Nearest Neighbors (kNNs), Random Forest (RFs), Multilayer Perceptron (MLP) neural networks, and Negative Selection Algorithms (NSAs) are shown in Table 7.

### 3.7. Evaluation of the Dataset with Selected Qualities Using Machine Learning Methods

**Figure 5:**
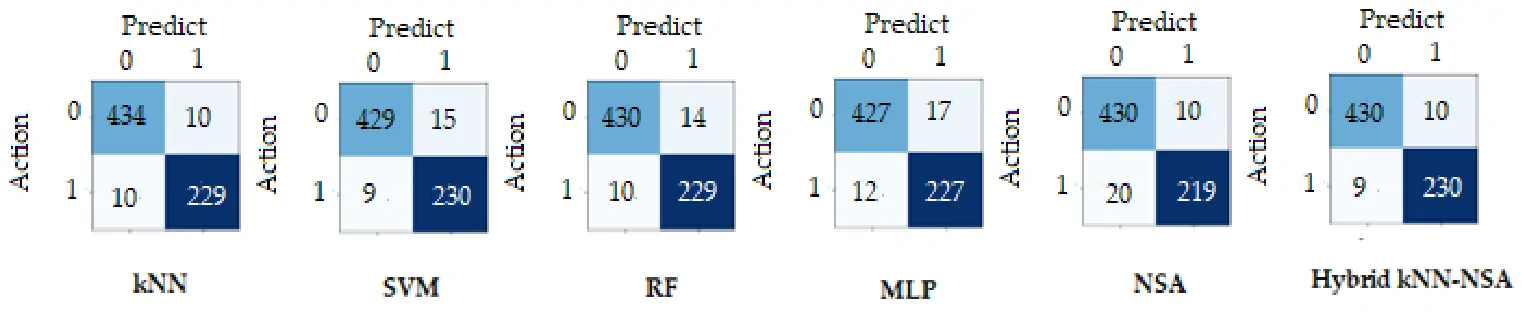
Confusion matrix of machine learning classifiers on the Breast Cancer Wisconsin dataset.

**Figure 6:**
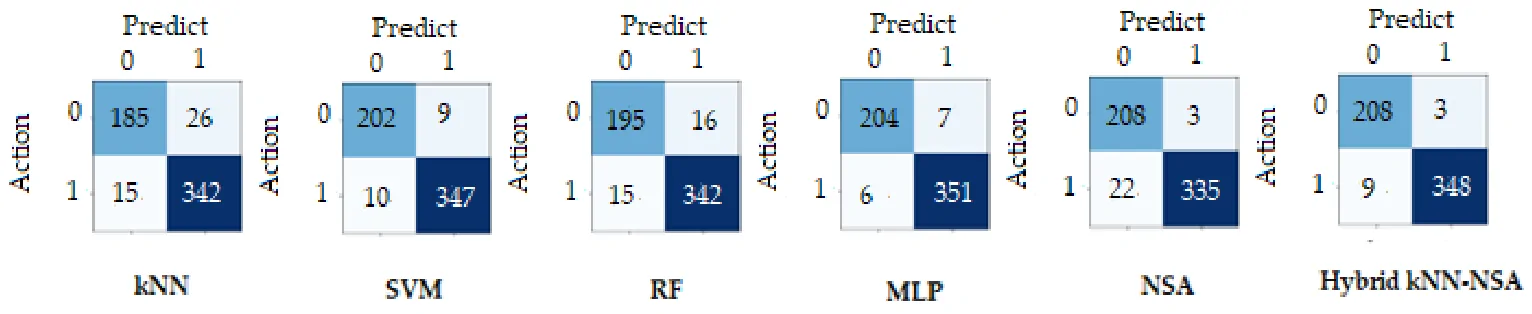
Confusion matrix of machine learning classifiers on the BreastEW dataset.

**Figure 7:**
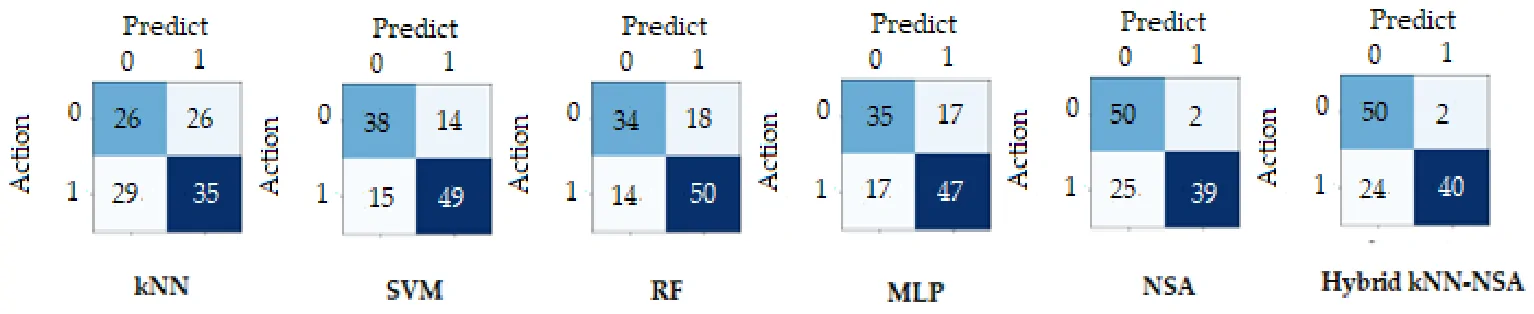
Confusion matrix of machine learning classifiers on the Breast Cancer Coimbra dataset.

**Figure 8:**
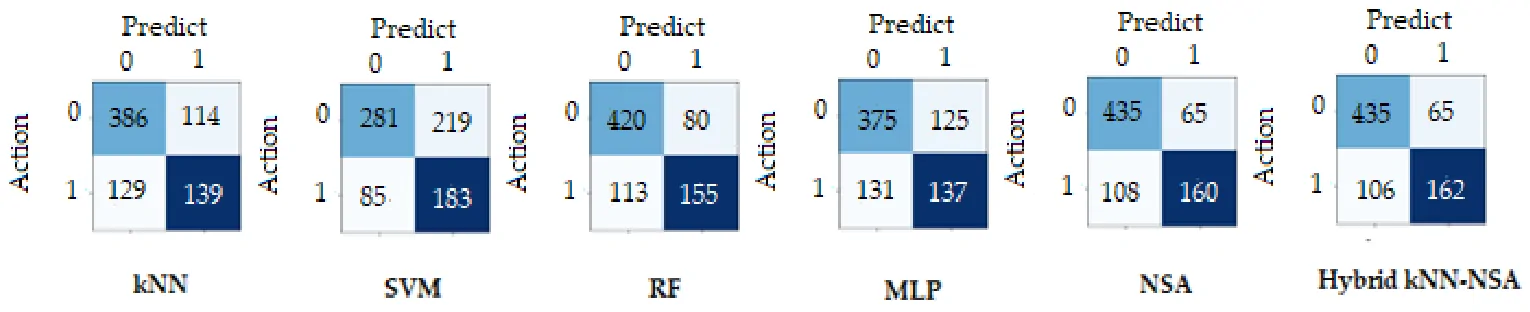
Confusion matrix of machine learning classifiers on the Pima Indians Diabetes dataset.

**Figure 9:**
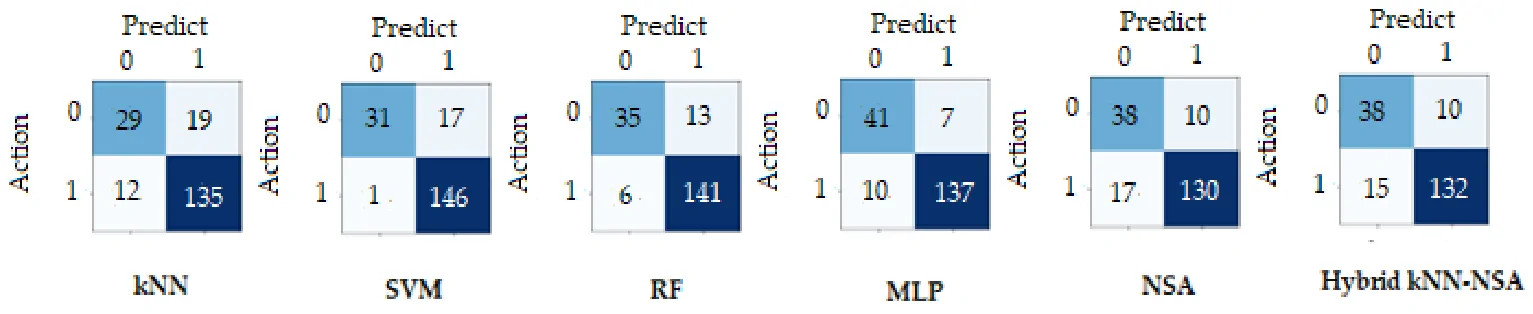
Confusion matrix of machine learning classifiers on the Parkinson Disease dataset.

**Figure 10:**
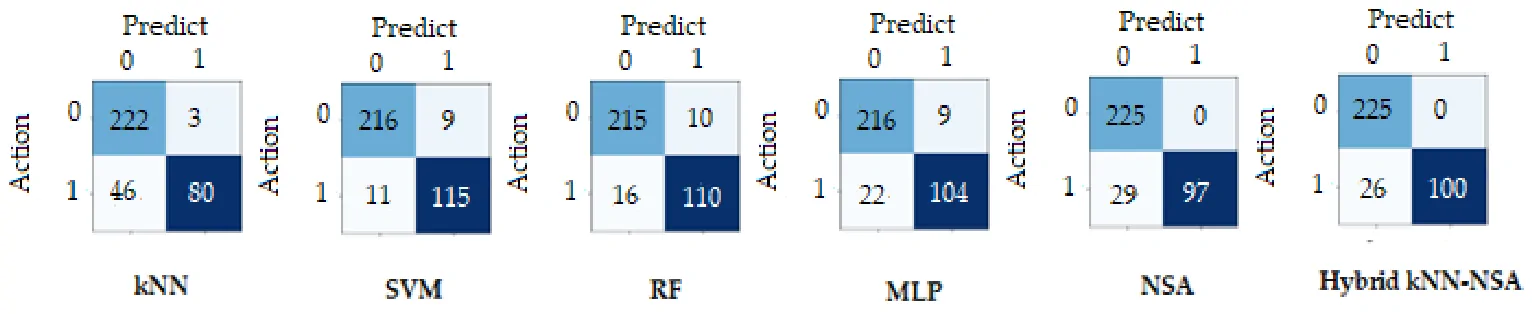
Confusion matrix of machine learning classifiers on the Ionosphere dataset.

**Figure 11:**
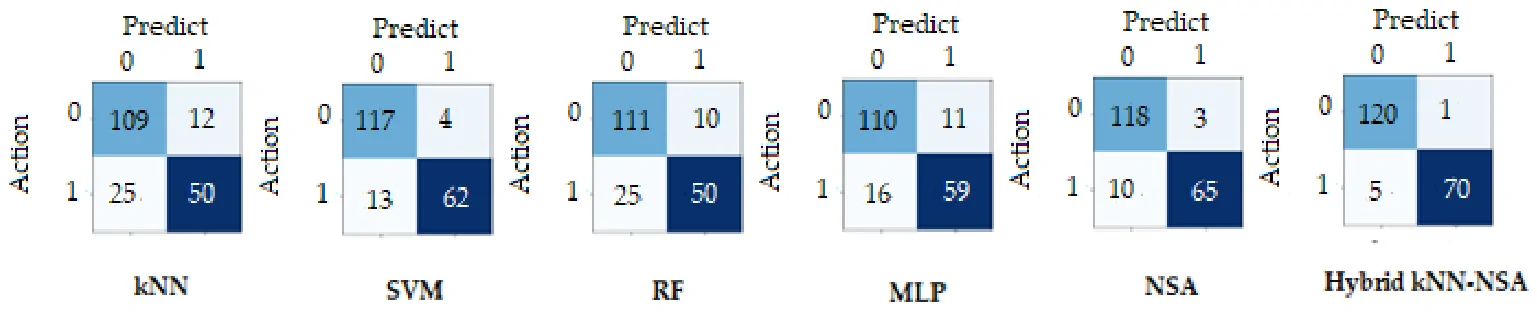
Confusion matrix of machine learning classifiers on the EpilepsyMRI(MWU35) dataset.

**Figure 12:**
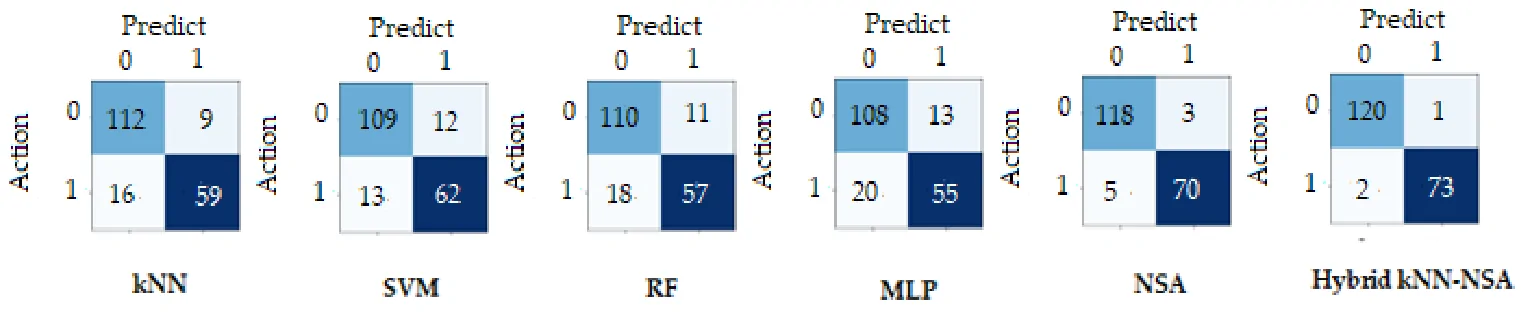
Confusion matrix of machine learning classifiers on the EpilepsyMRI(SSSO13) dataset.

In this study, the results obtained according to the machine learning performance criteria given in Table 1 are given in Table 8. The confusion matrix shows the performance of algorithms on datasets. Figure 5 shows the confusion matrix illustrating the performance of algorithms on the Breast Cancer Wisconsin dataset. Figure 6 shows the confusion matrix illustrating the performance of algorithms on the BreastEW dataset. Figure 7 shows the confusion matrix illustrating the performance of algorithms on the BreastCancerCoimbra dataset. Figure 8 shows the confusion matrix illustrating the performance of algorithms on the Pima Indians Diabetes dataset. Figure 9 shows the confusion matrix illustrating the performance of algorithms on the Parkinsons Disease dataset. Figure 10 shows the confusion matrix illustrating the performance of algorithms on the Ionosphere dataset. Figure 11 shows the confusion matrix illustrating the performance of algorithms on the EpilepsyMRI(MWU35) dataset. Figure 12 shows the confusion matrix illustrating the performance of algorithms on the EpilepsyMRI(SSSO13) dataset.

## 4. Discussion

The decision-making process for diagnosing epilepsy is quite difficult. Clinical evaluation and findings are important. EEG and MRI scans are used as diagnostic tools. In this study, we aimed to contribute to the decision-making process using machine learning methods and MRI data. The steps in the process are shown in Figure 1. MRI images were obtained retrospectively from the hospital archives with the approval of the ethics committee. These images were analyzed using FreeSurfer 3.0 software. Our goal was to statistically determine the significance between the control and experimental groups by performing brain volume analysis. Figure 2 shows the analysis steps of the FreeSurfer software. Using this software, volume values for 50 brain regions were obtained. These regions are shown in Table 3. The important question for us was which of these regions was significant for the patient and control groups. For this, the Mann–Whitney U test, a statistical analysis, was used. The results obtained are shown in Table 4. The 35 regions found to be statistically significant at the *p* < 0.05 level for both the control and experimental groups are indicated in red in Table 4. These regions constitute the features of our dataset.

The performance of machine learning algorithms in detecting epilepsy patients from a dataset consisting of 35 attributes was measured. The algorithms and parameters used are given in Table 7. 10-fold cross-validation was performed during the training phase. The results obtained are given in Table 8. Attribute reduction was used to improve patient detection performance. For this purpose, the SSSO algorithm was used. The attribute reduction performance of this algorithm is given in Table 5. Table 8 shows that the performance increased because of feature reduction.

The patient detection performance is also related to the machine learning algorithms used. To improve the performance of the NSA used for this purpose, a hybrid method has been developed. The performance of this method, called Hybrid kNN-NSA, is given in Table 8.

Looking at the data in Table 8, it can be seen that the developed hybrid method has also shown successful performance in terms of external validation.

The dataset created for this study to detect epilepsy demonstrated good performance in machine learning algorithms. Table 8 shows studies classifying epilepsy. As can be seen in the table, the best classification study was performed by Sahebzamani et al. [43]. However, because our goal was to detect epilepsy, our study yielded quite good results.

Table 8 shows the performance values of machine learning algorithms such as kNN, SVM, RF, MLP, NSA, and hybrid kNN-NSA on the EpilepsyMRI(MWU35) and EpilepsyMRI(SSSO13) datasets. An examination of the table reveals that the NSA performed best on the EpilepsyMRI(MWU35) dataset, as obtained using the Mann–Whitney U test. The NSA achieved a disease classification success rate of 93.37%. Its disease detection success rate was 95.59%. This shows that the NSA performs best with the EpilepsyMRI(MWU35) dataset. However, since our goal is to improve model performance, we aim to achieve this by both reducing the number of attributes in the dataset and developing a hybrid machine learning method. To achieve this, we applied the SSSO algorithm to the EpilepsyMRI(MWU35) dataset, reducing the number of features to 13. This created the EpilepsyMRI(SSSO13) dataset. When we examine the performance of the models on this dataset, we see an increase. The NSA also achieved a classification success rate of 95.92%. Detection success was measured at 95.89%. As can be seen here, the NSA significantly improved both the classification and detection success of the model. However, since our goal is to further improve the detection success of epilepsy, we are using the hybrid kNN-NSA algorithm we developed. With the hybrid optimized kNN-NSA algorithm, the detection success rate increased from 95.59% to 98.65%. When we compare these results with the results shown in Table 9, we can see that the 94% classification success rate achieved by Sahebzamani et al. [43] is high. This result demonstrates that the dataset generated with the selected features (regions) can be successful in detecting and classifying epilepsy. It also demonstrates the detection success of the optimized hybrid kNN-NSA for this problem.

**Figure 13:**
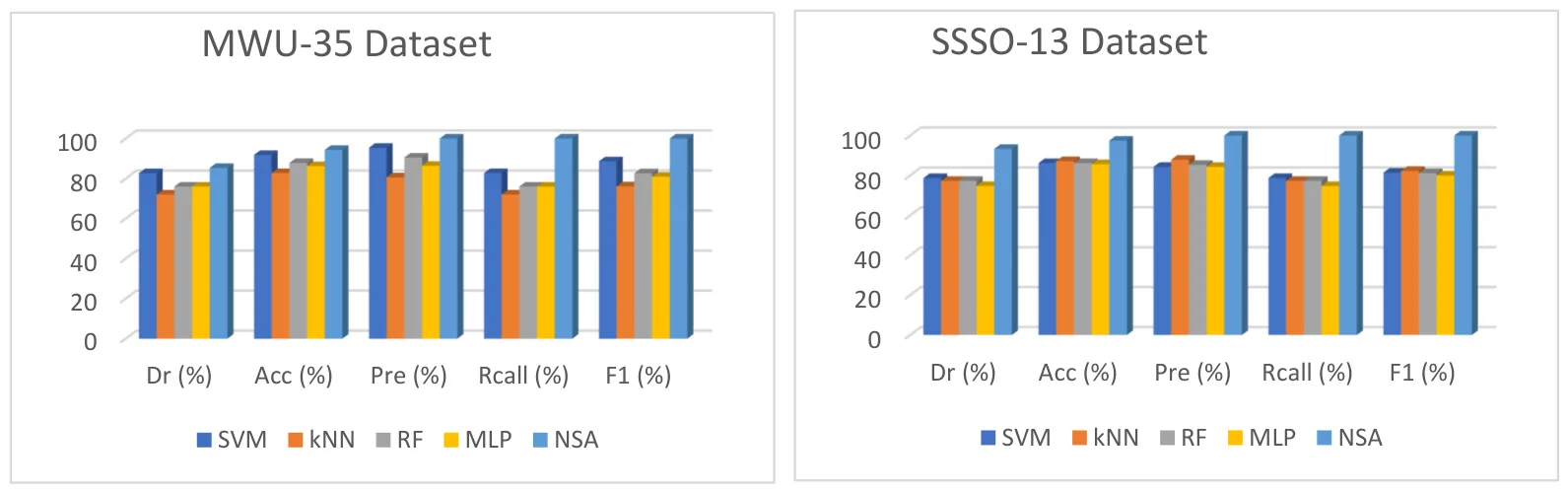
Performances of machine learning methods on EpilepsyMRI(MWU35) and EpilepsyMRI(SSSO13) datasets.

**Figure 14:**
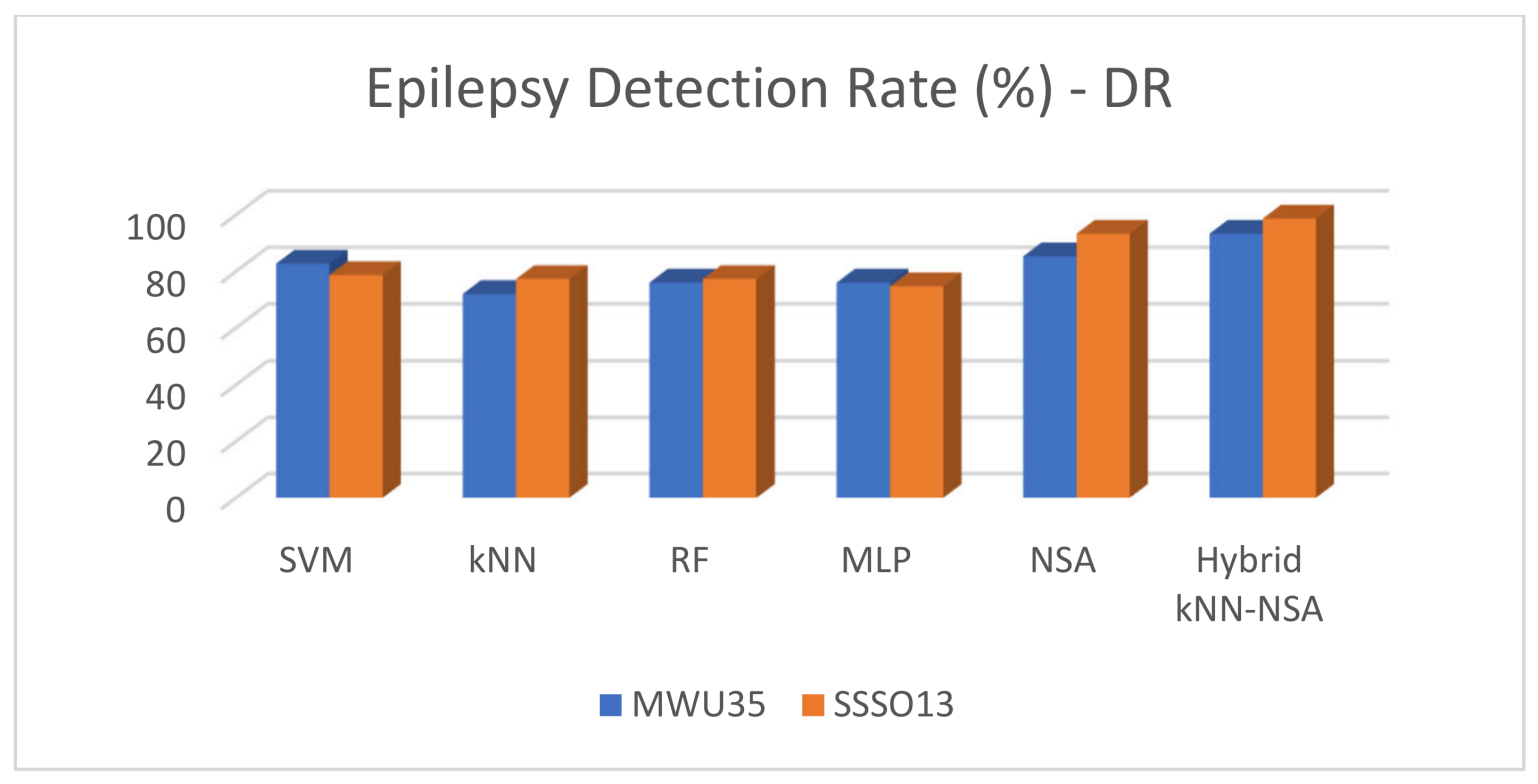
Comparison of the detection success (%) of the developed machine learning method.

To better understand the results obtained, they are graphically visualized in Figure 13 and Figure 14. As can be seen in the graph, the developed hybrid method offers good success in detecting epilepsy.

## 5. Conclusions

Diagnosing epilepsy requires observing the seizure, reporting these observations to the doctor, and conducting EEG, MRI, and any blood tests deemed necessary by the neurologist. EEG alone is not sufficient to establish or rule out the diagnosis of epilepsy. MRI can help identify the structural abnormality causing epilepsy, facilitate pre-surgical evaluation of drug-resistant epilepsy cases, and provide detailed visualization of anatomical structures. The sensitivity of MRI in detecting abnormalities depends on the pathological characteristics of the lesion, the MRI techniques used, and the experience of the interpreter. In human-based analysis of MRI images, computational methods are needed to reduce interobserver inconsistencies, the manpower required, and the analysis time. The rapid increase in patient numbers and the lack of sufficient manpower for image analysis is further increasing the need for such systems. For these reasons, the interest and need for image processing techniques in medical image analysis is increasing. Despite this long process, epilepsy is often difficult to diagnose and can sometimes be misdiagnosed.

Brain volume and surface area are important for many diseases, including autism, hyperactivity disorder, schizophrenia, multiple sclerosis, epilepsy, pre-term birth, fragile X syndrome, Tourette syndrome, and, in older adults, Alzheimer’s disease. Currently, stereological and morphological methods are used to estimate volume and surface area. Morphology is the branch of science that allows interpretations of three-dimensional specimens’ true three-dimensional properties from two-dimensional cross-sections. Using this technique, volume and surface area estimations can be made robustly and reliably on MRI images. There are limited resources available that calculate brain volume and surface area using morphological methods from MRI images of various anatomical structures.

The effectiveness of the negative selection algorithm used in patient detection systems has been clearly demonstrated by our research. During the patient detection phase, the time it takes for candidate detectors to achieve core set tolerance increases exponentially with the number of core sets. This occurs due to decreased efficiency and the production of many spare detectors. To address these issues, a novel patient detection framework based on negative selection theory is proposed.

The Negative Selection Algorithm (NSA) is an algorithm within Artificial Immune Systems that mimics how the body’s immune system creates T-cells that do not attack “self” cells. Learning is performed with “normal” data. It generates random detectors. If a detector matches the “normal” data, it is destroyed (negative selection). Detectors that do not match the normal data monitor newly arrived data. If a detector matches new data, it is marked as an “anomalous” (non-self) or an “anomaly.” Its strength is that it is very effective for anomaly detection and classification. Its weakness is that it is difficult to produce efficient detectors. Randomly generating detectors can leave large gaps in the search space (non-self-space) or lead to the unnecessary creation of too many detectors (computational overhead). This algorithm can create a very powerful anomaly detection model, especially in cybersecurity (network intrusion detection), industrial systems (fault detection) or complex biological data (cancer detection).

Our study surpassed the 94% classification accuracy achieved by Sahebzamani et al. [43]. The results of this study demonstrate that a dataset created with the selected features (regions) can be successful in detecting and classifying epilepsy. It also demonstrates the detection success of the optimized hybrid kNN-NSA for this problem.

We also believe that it may be effective in diagnosing epilepsy in regions not previously reported in the literature, such as WM hypointensities, ventral DC, and choroid plexus. We believe that performing different segmentation and volume analyses on these regions could contribute to the literature.

The literature review reveals that there are very few studies classifying brain volume and surface area in epilepsy. We believe that this study will contribute to the literature.

## Figures and Tables

**Table 1:** Precision (PRE) to measure the accuracy of the detections.

Performance Metric	Formula
Precision (Prec)	$\frac{TP}{TP+FP}$
Recall (Recall)	$\frac{TP}{TP+FN}$
F1-score	$\frac{2*Prec*Recall}{Prec+Recall}$
Accuracy (Acc)	$\frac{TP+TN}{TP+FP+TN+FN}$

**Table 2:** Demographic and intracranial volume (ICV) of epilepsy disease and healthy control groups.

Variables	Healthy Control (HC) (*n* = 121)	Epilepsy Disease (ED) (*n* = 75)
Age Mean ± SD	35.2 ± 6.5	34.8 ± 6.8
Gender (Female/Male) Number (%)	58 (%47.93)/63 (%52.07)	35 (%46.7)/40 (%53.3)
Intracranial Volume (ICV) Mean ± SD (cm^3^)	1483 ± 113	1476 ± 120

**Table 3:** Regions extracted from the cortical and subcortical structure of the brain.

Extracted Brain Regions			
1	Left hemisphere cortical gray matter	26	Left-vessel
2	Right hemisphere cortical gray matter	27	Left-choroid-plexus
3	Total cortical gray matter	28	Right-Lateral-Ventricle
4	Left hemisphere cortical white matter	29	Right-Inf-Lat-Vent
5	Right hemisphere cortical white matter	30	Right-Cerebellum-White-Matter
6	Total cortical white matter	31	Right-Cerebellum-Cortex
7	Subcortical gray matter	32	Right-Thalamus-Proper
8	Total gray matter	33	Right-Caudate
9	Estimated Total Intracranial	34	Right-Putamen
10	Left-Lateral-Ventricle	35	Right-Pallidum
11	Left-Inf-Lat-Vent	36	Right-Hippocampus
12	Left-Cerebellum-White-Matter	37	Right-Amygdala
13	Left-Cerebellum-Cortex	38	Right-Accumbens-area
14	Left-Thalamus	39	Right-VentralDC
15	Left-Caudate	40	Right-vessel
16	Left-Putamen	41	Right-choroid-plexus
17	Left-Pallidum	42	5th-Ventricle
18	3rd-Ventricle	43	WM-hypointensities
19	4th-Ventricle	44	non-WM-hypointensities
20	Brain-Stem	45	Optic-Chiasm
21	Left-Hippocampus	46	CC_Posterior
22	Left-Amygdala	47	CC_Mid_Posterior
23	CSF	48	CC_Central
24	Left-Accumbens-area	49	CC_Mid_Anterior
25	Left-VentralDC	50	CC_Anterior

**Table 4:** Independent samples Mann–Whitney U test analysis results (*p* < 0.05).

	Region	Sig.		Region	Sig.
1	Left hemisphere cortical gray matter	0.136	26	Left-vessel	0.197
2	Right hemisphere cortical gray matter	0.84	27	Left-choroid-plexus	0.001
3	Total cortical gray matter	0.85	28	Right-Lateral-Ventricle	0.002
4	Left hemisphere cortical white matter	0.227	29	Right-Inf-Lat-Vent	0
5	Right hemisphere cortical white matter	0.144	30	Right-Cerebellum-White-Matter	0.001
6	Total cortical white matter	0.159	31	Right-Cerebellum-Cortex	0.007
7	Subcortical gray matter	0	32	Right-Thalamus-Proper	0.863
8	Total gray matter	0.001	33	Right-Caudate	0
9	Estimated Total Intracranial	0.011	34	Right-Putamen	0
10	Left-Lateral-Ventricle	0	35	Right-Pallidum	0
11	Left-Inf-Lat-Vent	0	36	Right-Hippocampus	0
12	Left-Cerebellum-White-Matter	0.069	37	Right-Amygdala	0
13	Left-Cerebellum-Cortex	0.001	38	Right-Accumbens-area	0
14	Left-Thalamus-Proper	0.038	39	Right-VentralDC	0.001
15	Left-Caudate	0.119	40	Right-vessel	0.72
16	Left-Putamen	0	41	Right-choroid-plexus	0
17	Left-Pallidum	0	42	5th-Ventricle	0.757
18	3rd-Ventricle	0.148	43	WM-hypointensities	0
19	4th-Ventricle	0.001	44	non-WM-hypointensities	0
20	Brain-Stem	0	45	Optic-Chiasm	0
21	Left-Hippocampus	0	46	CC_Posterior	0
22	Left-Amygdala	0	47	CC_Mid_Posterior	0.004
23	CSF	0.159	48	CC_Central	0.006
24	Left-Accumbens-area	0	49	CC_Mid_Anterior	0.225
25	Left-VentralDC	0.01	50	CC_Anterior	0

**Table 5:** Performance comparison of various optimization algorithms on the epilepsy dataset [34].

	Acc	Error	Sensitive	Spec	Prec	F1
SSSO	0.8712	0.1288	0.9551	0.7375	0.8573	0.9036
GNDO	0.8638	0.1362	0.9301	0.7586	0.8644	0.8961
SMA	0.8659	0.1341	0.9336	0.7571	0.8644	0.8977
EO	0.8638	0.1362	0.9219	0.7711	0.8698	0.8951
MRFO	0.8596	0.1404	0.9268	0.7521	0.8601	0.8922
ASO	0.8599	0.1401	0.9401	0.7311	0.8520	0.8939
HHO	0.8384	0.1616	0.9050	0.7282	0.8469	0.8750
HGSO	0.8547	0.1453	0.9254	0.7432	0.8588	0.8908
PFA	0.8702	0.1298	0.9567	0.7318	0.8547	0.9028
PRO	0.8731	0.1269	0.9468	0.7546	0.8652	0.9041
SS	0.8287	0.1713	0.8950	0.7218	0.8445	0.8690
GA	0.8649	0.1351	0.9467	0.7336	0.8546	0.8983
PSO	0.8605	0.1395	0.9287	0.7525	0.8628	0.8946
ACO	0.8598	0.1402	0.9421	0.7286	0.8514	0.8945
DE	0.8585	0.1415	0.9183	0.7629	0.8650	0.8909
ABC	0.8662	0.1338	0.9500	0.7321	0.8545	0.8997
GWO	0.8596	0.1404	0.9169	0.7679	0.8675	0.8915
WOA	0.8618	0.1382	0.9253	0.7604	0.8660	0.8947

**Table 6:** Features obtained with the SSSO algorithm.

	Brain Regions (Features)
1	Subcortical gray matter
2	Left-Cerebellum-Cortex
3	Left-Thalamus
4	Left-Putamen
5	Left-VentralDC
6	Left-choroid-plexus
7	Right-Putamen
8	Right-Hippocampus
9	Right-VentralDC
10	Right-choroid-plexus
11	WM-hypointensities
12	CC_Posterior
13	CC_Central

**Table 7:** Parameters used in machine learning algorithms.

SVM	kNN	RF	MLP	NSA	Hybrid kNN-NSA
Kernel = RBF	Number of neighbors = 3	Size of each bag = 7	Learning rate = 0.003	R_s_: 0.33	Optimal k = 3
Cost(C) = 150	Distance function = Euclidean	Max depth = 0	Momentum = 0.9	Detector: 5000	Optimal threshold_P = 0.2
		No. of trees = 20	Hidden layers = 35		

**Table 8:** Percentage performance of classifiers according to datasets.

Datasets	Method	Acc	F1	Prec	Recall
BreastCancerWisconsin (UCI)	kNN	97.07	95.81	95.81	95.81
	SVM	96.48	95.04	93.87	96.23
	RF	96.48	95.02	94.23	95.81
	MLP	95.75	94.00	93.03	94.97
	NSA	95.58	93.58	95.63	91.63
	Hybrid kNN-NSA	97.20	96.03	95.83	96.23
BreastEW (UCI)	kNN	92.78	94.34	92.93	95.79
	SVM	96.65	97.33	97.47	97.19
	RF	94.54	95.66	95.53	95.79
	MLP	97.71	98.18	98.04	98.31
	NSA	95.59	96.40	99.11	93.83
	Hybrid kNN-NSA	97.88	98.30	99.14	97.47
BreastCancerCoimbra (UCI)	kNN	52.58	56.00	57.37	54.68
	SVM	75.00	77.16	77.77	76.56
	RF	72.41	75.75	73.52	78.12
	MLP	70.68	73.43	73.43	73.43
	NSA	76.72	74.28	95.12	60.93
	Hybrid kNN-NSA	77.58	75.47	95.23	62.50
Pima Indians Diabetes	kNN	68.35	53.35	54.94	51.86
	SVM	60.41	54.62	45.52	68.28
	RF	74.86	61.63	65.95	57.83
	MLP	66.66	51.69	52.29	51.11
	NSA	77.47	64.90	71.11	59.70
	Hybrid kNN-NSA	77.73	65.45	71.36	60.44
Parkinsons Disease (Oxford)	kNN	84.10	89.70	87.66	91.83
	SVM	90.76	94.19	89.57	99.31
	RF	90.25	93.68	91.55	95.91
	MLP	91.28	94.15	95.13	93.19
	NSA	86.15	90.59	92.85	88.43
	Hybrid kNN-NSA	87.17	91.34	92.95	89.79
Ionosphere (Johns Hopkins University)	kNN	86.03	76.55	96.38	63.49
	SVM	94.30	92.00	92.74	91.26
	RF	92.59	89.43	91.66	87.30
	MLP	91.16	87.02	92.03	82.53
	NSA	91.73	86.99	100.00	76.98
	Hybrid kNN-NSA	92.09	87.82	100.00	78.29
EpilepsyMRI (MWU35)	kNN	81.10	73.30	80.60	66.70
	SVM	91.30	87.90	93.90	82.70
	RF	82.10	74.10	83.30	66.70
	MLP	86.20	81.40	84.30	78.70
	NSA	93.37	90.91	86.67	95.59
	Hybrid kNN-NSA	96.94	95.89	93.33	95.89
EpilepsyMRI (SSSO13 [45])	kNN	87.20	82.50	86.80	78.70
	SVM	87.20	83.20	83.80	82.70
	RF	85.20	79.70	83.80	76.00
	MLP	83.20	76.90	80.90	73.30
	NSA	95.92	94.60	99.33	95.89
	Hybrid kNN-NSA	98.47	98.00	97.33	98.65

**Table 9:** An overview of the most recent studies in the diagnosis of epilepsy.

Study	Method	Number of Features	Dataset	Acc (%)
Keihaninejad et al. [27]	SVM	83	80 Epilepsy 28 Healthy	89
Canto-Rivera et al. [35]	SVM	10	17 Epilepsy 19 Healthy	88.9
Rudie et al. [36]	SVM	20	85 Epilepsy 84 Healthy	81
Kodipaka et al. [37]	SVM	762	31 Epilepsy 23 Healthy	80.65
Zhou et al. [42]	SVM	34	74 Epilepsy 74 Healthy	84
Sahebzamani et al. [43]	SVM	36	10 Epilepsy 7 Healthy	94
Beheshti et al. [55]	SVM	136	39 Epilepsy 24 Healthy	87.30
Nguyen et al. [56]	CNN	36	63 Epilepsy 259 Healthy	78
Princich et al. [57]	RF	20	57 Epilepsy 61 Healthy	90.7
Huang et al. [58]	CNN-SVM	36	59 Epilepsy 70 Healthy	90.8
Proposed Method [34]	kNN-NSA	13	75 Epilepsy 121 Healthy	98.45

## Data Availability

The EpilepsyMRI dataset generated during and/or analyzed during the current study is not publicly available due to privacy concerns but is available from the corresponding author upon reasonable request. The EpilepsyMRI dataset consists of the data we originally obtained. For this dataset, DICOM-format brain MRI images from the neurology archive of Aksaray University Aksaray Training and Research Hospital MR Image Service were used.
